# Impact of Early Diagnosis and Immunosuppressive Therapy on Giant Cell Myocarditis Outcomes: A Review

**DOI:** 10.3390/life16040575

**Published:** 2026-04-01

**Authors:** Nilima Rajpal Kundnani, Abhijit Kumar, Abhinav Sharma, Berceanu Vaduva Marcel Mihai, Cristina Diana Ardelean, Lucretia Marin-Bancila, Mihaela Valcovici, Codrina Levai, Adela Iancu, Ciprian Ilie Rosca

**Affiliations:** 1 Department VI-Cardiology, “Victor Babes” University of Medicine and Pharmacy, 300041 Timisoara, Romania; 2 Research Centre of Timisoara, Institute of Cardiovascular Diseases, “Victor Babes” University of Medicine and Pharmacy, 300041 Timisoara, Romania; 3 Department of Anaesthesiology, All India Institute of Medical Sciences (AIIMS), Kalyani 741245, India; 4 Doctoral School, “Victor Babes” University of Medicine and Pharmacy, 300041 Timisoara, Romania; 5 Department XVI-Orthopedics-Traumatology, Urology and Medical Imaging, “Victor Babes” University of Medicine and Pharmacy Timisoara, Eftimie Murgu Square No. 2, 300041 Timisoara, Romania; 6 Faculty of Medicine, “Victor Babes” University of Medicine and Pharmacy Timisoara, Eftimie Murgu Square No. 2, 300041 Timisoara, Romania; 7 Discipline of Medical Communications, Department 2-Microscopic Morphology, “Victor Babes” University of Medicine and Pharmacy, 300041 Timisoara, Romania; 8 Research Center for Medical Communication, “Victor Babes” University of Medicine and Pharmacy Timisoara, Eftimie Murgu Square No. 2, 300041 Timisoara, Romania; 9 IInd Family Medicine Department, “Carol Davila” University of Medicine and Pharmacy, 020021 Bucharest, Romania; 10 Department V, Internal Medicine I—Discipline of Medical Semiology I, Center of Advanced Research in Cardiology and Hemostasology, “Victor Babes” University of Medicine and Pharmacy, 300041 Timisoara, Romania

**Keywords:** giant cell myocarditis, early diagnosis, immunosuppressive therapy, cardiogenic shock, mechanical support mechanisms, histological examination

## Abstract

Background: Giant cell myocarditis (GCM) is a rare condition with an incompletely understood immune pathogenesis, characterized by inflammatory damage to the myocardium and the presence of multinucleated giant cells on histopathological examination. The frequently fulminant and severe course requires rapid intervention for a correct diagnosis and the initiation of immunosuppressive therapy, which is often life-saving. Materials and methods: This article contains information from observational studies and case reports, systematically collected from prestigious publications such as JACC, NEJN, ESC, JCC, Heliyon, and Cureus found in the PubMed and ClinicalTrials.gov databases. Thus, 25 patients diagnosed with giant cell myocarditis between March 2019 and May 2025 were analyzed, with a focus not only on the initial clinical evolution, mortality incidence, and the need for heart transplantation but also on the incidence of major complications such as cardiogenic shock and malignant rhythm and conduction disorders refractory to drug treatment. These parameters were studied according to certain intrinsic factors that cannot be influenced, such as age at onset, gender, and associated pathology of the patient, as well as extrinsic factors that can be influenced, such as the time of diagnosis and the start of immunosuppressive therapy. The results obtained were compared with those in the literature from previous years, considering the limitations of the current study. Results: The selected patients were 13 women (52%) and 12 men (48%), mostly from the US and Japan, aged between 22 and 76 years, with an average age of 44.92 years. An associated autoimmune pathology was found in 40% of patients in this group, and previous cardiovascular pathology in 28%. Only 8% had a history of GCM. The clinical onset of new-onset heart failure, refractory to usual therapy, with progressive dyspnea as the cardinal symptom was found in 12 patients, representing 48% of cases; palpitations as an expression of rhythm or conduction disorders were found in five patients, representing 20%; precordial discomfort to precordial pain accompanied or not by ST-T segment changes was present in four patients, representing 16%; and general signs and symptoms or those of other organs were present in three (12%) cases. The diagnosis was made by histological examination of the biopsy fragment obtained by endomyocardial biopsy or from the myocardial fragment obtained during the implantation of mechanical cardiovascular support devices and, less frequently, on the explanted heart and at autopsy. In terms of progression, of the 25 patients, four (16%) died, four (16%) required heart transplantation, and 16 (64%) had a severe progression with cardiogenic shock, which required mechanical circulatory support in 11 (44%) cases. The outcome was mainly influenced by the early diagnosis and administration of immunosuppressive medication, but also by the age of the patients and associated chronic diseases. Conclusions: Giant cell myocarditis is a serious condition that, in the absence of rapid diagnosis and appropriate immunosuppressive therapy, has a fulminant, often fatal course. Clinical suspicion of giant cell myocarditis remains important in the initial diagnosis. Raising this suspicion, together with modern and improved paraclinical investigations compared to previous years, has led to faster diagnosis and administration of immunosuppressive therapy in this pathology. Histological examination remains the gold standard for final diagnosis, but it should be noted that it may be non-diagnostic. In the face of a strong suspicion of giant cell myocarditis, the best approach is to start immunosuppressive therapy and monitor the patient’s progress. Immunosuppressive treatment remains decisive in influencing the evolution of this condition, both through prompt administration and through the adaptation of therapeutic regimens to the evolution of patients. A more detailed understanding of the immune-mediated pathogenesis of GCM and the identification of clinical risk factors for unfavorable short- and long-term outcomes may enable earlier risk stratification and the development of more targeted, individualized therapeutic strategies.

## 1. Introduction

Myocarditis is an inflammatory disease of the myocardial tissue, with multiple etiologies, defined by well-established histological, immunological, and immunohistochemical criteria that cause varying degrees of damage to the structure and function of the heart [1,2,3,4,5]. The association between myocarditis and mechanical and/or electrical cardiac dysfunction is part of the group of cardiomyopathies [1].

Being an extremely heterogeneous group, myocarditis has been classified using several criteria [1,2], the most used being etiopathogenic, clinical-evolutionary, and histological.

This study will address one of the forms of myocarditis characterized by histopathological criteria, giant cell myocarditis, which is notable for its frequently fulminant course and a mortality rate of nearly 100% in the absence of immunosuppressive therapy.

Giant cell myocarditis (GCM) is an inflammatory condition of the myocardium characterized histopathologically by the presence of multinucleated giant cells along with a nonspecific inflammatory infiltrate consisting of lymphocytes, histiocytes, eosinophils, and non-ischemic myocyte necrosis [3,4,6,7]. The etiopathogenesis of this disease is not fully understood, but it is believed to be immune-mediated, involving TCD4+ lymphocytes in particular [6,7,8,9,10,11].

This form of myocarditis was first described in a young man with acute heart failure by Saltykow in 1905 [6,7]. It is a rare disease that commonly affects young and middle-aged adults with no gender predominance and a very heterogeneous clinical presentation. Thus, often fulminant heart failure resistant to usual therapy and recurrent and refractory rhythm and conduction disorders, with cardiogenic shock and sudden death, are the most common clinical presentations [1,2,3,4,6].

Retrospective studies have demonstrated an association of this form of myocarditis with autoimmune diseases in approximately 19–20% of cases [7,11].

Due to the heterogeneity of the clinical presentation of GCM, its fulminant course, often complicated by cardiogenic shock and even sudden death, the diagnosis of this disease is often challenging. Furthermore, the difficulty in diagnosing this pathology also stems from the fact that a definitive diagnosis is made through the histological study of a biopsy fragment obtained by several methods, namely endomyocardial biopsy puncture, resection of a fragment from the left ventricular apex during the implantation of mechanical cardiovascular support devices, and histological examination of the explanted heart [7,11,12].

Compared to the 1980s, when diagnosis was mainly made at autopsy, there has been an increase in survival rates for this disease. There have been multiple factors influencing the decrease in mortality from this disease, namely [7,10,13,14,15]:

- The possibility of making a faster diagnosis due to the improvement of the main paraclinical investigations (ETT, coronary angiography, cardiac catheterization and CMI, CT, including imaging guidance of endomyocardial biopsy-EMB) and, implicitly, the faster administration of this life-saving therapy.
- The expansion of knowledge about the pathogenesis of this disease, which has led to more effective immunosuppressive therapy, both through combinations of drugs with immunosuppressive effects through different mechanisms and through the use of latest-generation immunosuppressive drugs.
- Improvements in mechanical cardiovascular support devices and a decrease in the incidence of mortality in the evolution of cardiogenic shock.
- Effective monitoring at discharge and the use of devices such as ICDs and pacemakers in conjunction with immunosuppressive therapy have led to a decrease in mortality from rhythm and conduction disorders.

The article aims to demonstrate the importance of early diagnosis of giant cell myocarditis and, implicitly, of administering immunosuppressive therapy in the evolution of this disease.

## 2. Materials and Methods

For this study, data were collected from the specialized literature indexed in PubMed (National Library of Medicine, Bethesda, MD, USA) and ClinicalTrials.gov (U.S. National Library of Medicine, Bethesda, MD, USA), including publications in major medical journals such as JACC, NEJM, ESC publications, JCC, Heliyon, and Cureus. This work represents a narrative review of published case reports and case series rather than a formal systematic review.

Data were collected from observational studies and case reports published between March 2019 and May 2025; thus, 25 patients diagnosed with giant cell myocarditis during this period were selected and analyzed.

In these patients, the incidence of mortality, the need for heart transplantation, and the incidence of major complications (cardiogenic shock and refractory rhythm and conduction disorders) were analyzed according to certain intrinsic and extrinsic factors. Thus, the influence of the following factors on the evolution of these patients was monitored:

- Intrinsic (uninfluential) factors: patient age, gender, and associated diseases;
- Extrinsic (influential) factors: the time of diagnosis and the start of immunosuppressive therapy.

In order to obtain an overview of the evolution of this condition over the last five years, the general characteristics of the affected population, the clinical forms of onset, the main paraclinical investigations used for diagnosis, and the therapeutic approach were also presented.

The results obtained were compared with previous studies, taking into account the limitations of this study. The comparisons aim to highlight the changes in recent years in the fate of patients diagnosed with GCM, driven by improvements in paraclinical investigations, a multidisciplinary approach, and the use of modern cardiac support equipment.

The importance of improving the efficiency of immunosuppressive therapy, both through rapid administration and through the adaptation of therapeutic regimens according to the evolution of the disease, is also mentioned.

Broadening the knowledge of pathogenesis and risk factors for this disease, as well as for an unfavorable evolution in the course of the disease in the short or long term, will allow the development of concrete and effective therapeutic regimens.

The ultimate goal is to emphasize the continuing importance of early diagnosis and administration of immunosuppressive therapy in the evolution of this pathology.

## 3. Results

### 3.1. General Characteristics of Patients in the Study Group

Of the 25 patients selected, 13 were women (52%) and 12 were men (48%), mainly from the US, Japan, Canada, China, Germany, the Czech Republic, and Honduras (who had emigrated to the US 15 years ago).

The patients’ ages ranged from 22 to 76 years, with an average age of 45.92 years for the group (Table 1).

### 3.2. Personal Medical History of the Study Group (Table 1)

Significant pathology prior to giant cell myocarditis was present in 14 of the 25 patients, i.e., approximately 56%.

- Autoimmune pathological history was the most frequent, occurring in 10 patients, representing 40% of this group. Furthermore, some patients had multiple autoimmune conditions or presented with multiple pathologies of different etiology (Table 1).
- Cardiovascular pathological antecedents were found in seven patients, i.e., 28%, of whom two (8%) had a history of giant cell myocarditis, with one patient undergoing heart transplantation for this disease (Table 1).
- Previously operated neoplastic diseases were found in two (8%) patients, and acquired common variable immunodeficiency and acute pyelonephritis were found in one (4%) patient each.

### 3.3. Forms of Onset of Giant Cell Myocarditis in the Study Group (See Also Table 2)

The onset of giant cell myocarditis was predominantly insidious in 15 (60%) of the 25 patients. The main symptoms were:

1. Progressive dyspnea, which was the most frequent symptom, varying in intensity from mild intermittent dyspnea to orthopnea-NYHA IV [38]; it was encountered in 12 (48%) patients, variably accompanied by other symptoms such as fever, decreased exercise capacity, cough, fatigue, and diarrhea. Accompanying symptoms such as precordial pressure and even precordial pain required differential diagnosis with acute coronary syndrome (ACS) and sometimes with pulmonary embolism and acute pneumopathies [23]. In most cases, dyspnea was the expression of a primarily mechanical cardiac impairment and, more rarely, the expression of electrical disturbances (AVB, BRD, various arrhythmias), while in other cases the mechanisms were complex from the outset (see Table 2).
2. Palpitations were encountered in five (20%) cases, with secondary symptoms of vertigo or chest tightness [19]. They were the clinical expression of various rhythm disorders ranging from bigeminal ventricular extrasystoles to sustained ventricular tachycardia [18,19,32] or SSS conduction disorders [38].
3. Precordial pain of varying intensity, ranging from precordial discomfort to well-defined precordial pain, was encountered in 4 (16%) cases [27,31,33,36]. This was sometimes accompanied by changes in the ST segment and T wave [31,33], requiring differential diagnosis with acute coronary syndrome, while at other times it was the clinical expression of reduced blood flow during ventricular arrhythmias [36]. Secondary symptoms were fever [27,33], dyspnea [33], signs of hypoperfusion, and shock [27].
4. Other modes of onset were the presence of a viral prodromal syndrome [34]; symptoms related to pre-existing pathology such as myalgia, muscle weakness, diplopia, and expression of polymyositis, which dominated the onset and initial clinical picture, leading to delays in diagnosis [16]; or signs and symptoms of other organs, which are difficult to diagnose, such as nausea and vomiting interpreted as acute dehydration syndrome [21].
5. Signs of hyperperfusion and shock requiring cardiac support with inotropic medication and/or mechanical cardiovascular support were encountered on the first day of hospitalization in 5 (20%) patients (Table 3).

**Table 2:** Correlations between initial symptoms and paraclinical investigations.

Case No./(Bibliography)	Onset	EKG	ETT	Other Investigations Carried Out
1 [16]	Relapse GCM Cardiogenic shock		LVEF = 15%	
2 [17]	Insidious fever dyspnea	ST Mild ST elevation V1–V2	Severe hypokinesia of the apex and left ventricular wall Diffuse left ventricular edema EF = 50%	CAG
3 [12]	Insidious progressive dyspnea NYHA IV	TS + RBBB	Severe hypokinesia of the LV LVEF = 30%	CXR
4 [18]	Sudden Vertigo + Palpitations	Ventricular tachycardia	Severe biventricular dysfunction with low DC	MRI CAG insignificant PET-CT
5 [19]	Precordial pressure Palpitations	Chronic TSV	Normal biventricular function LVEF = 60% Mild RM Mild HTP	MRI
6 [20]	Insidious: - Progressive dyspnea accentuated by exertion and clinostatism	ST	global LV hypokinesia LVEF = 30–35%	CAG CXR MRI
7 [21]	Insidious Nausea & vomiting			
8 [22]	Feeling of fainting Progressive	Complete Atrioventricular (AV) block	TTE normal	CXR Cardio CT CC
9 [23]	Insidious With dyspnea Acute precordial discomfort	ST + RBBB	Severely affected LV function LVEF = 26% moderat\sever right ventrcular systolic dysfunction changes and moderate/severe functional MR	CT
10 [24]	Insidious palpitations	Sick sinus syndrome (SSS)	AD tumor formation 55 × 30 mm, bi-atrial dilatation Mild/moderate RT Small amount of fluid in the pleural cavity LVEF = 67%	Cardiopulmonary CT
11 [25]	Insidious dyspnea Feeling of pressure in the chest	ST elevation in V2–V3	Dilated LV with LVEF = 10%	CAG CC
12 [26]	Insidious dyspnea vertigo progressive dyspnea	LBBB Atrioventricular block grade 1 Holter discharged	No changes in kinetics LVEF = 55%	MRI LGE non dgs
13 [27]	fever precordial pain signs of shock and multiple organ dysfunction		Severe reduction in LVEF = 16%	
14 [38]	Insidious severe dyspnea HF decompensation refractory to treatment	ST	Global systolic dysfunction of the LV LVEF = 13%	
15 [28]	Insidious reduced exercise capacity, dyspnea	RS + RBBB	Mild reduction in LVEF = 48% with apical and septal hypokinesia Dilated RV with reduced function EF = 47%	MRI LEG CXR PET-CT
16 [29]	Insidious progressive dyspnea		LVEF = 30–35%	CMR Nondgs CAG
17 [30]	Insidious Fever Chills Diarrhea Worsening dyspnea	Micro voltage evolution refractory arrhythmias and multiple organ dysfunction	with previous non-ischemic CMD EF = 35%	MRI CAG CC - low Coxsackie BDC antibody titer
18 [31]	Precordial discomfort	Nonspecific and diffuse ST-T elevation	LV thickening low LV contraction	CAG
19 [32]	Patient CT scan Shock on ICD Prior to shock Presyncope Palpitations	sustained monomorphic VT on implanted defibrillator		
20 [33]	Precordial pressure Dyspnea Fever	RS Right deviation of Neg T inferior derivation RBBB	Mild hypokinesia reg inferoseptal LV, small pericardial effusion LVEF = 55%	MRI CC CAG
21 [34]	Insidious dyspnea precordial pressure	ST AXIS deviation	Concentric hypertrophy VS small-medium pericardial effusion LVEF 55%	CAG CC
22 [34]	Insidious viral prodrome cough fatigue joint pain malaise fever	ST RBBB	Global hypokinesia LVEF = 20–25%	CC
23 [35]	Insidious back pain myalgia of the lower limbs	ST ST elevation in DI, aVL, V3–V6 ST elevation in aVR	Slightly decreased LVEF with wall thickening	T2-weighted MRI CAG CT
24 [36]	Feeling of pressure in the chest	Refractory ArV TV evolution	Severe depression of both ventricles LVEF = 10%	CXR
25 [37]	Dyspnea, fever nausea, vomiting, and diarrhea	RS	LVEFS = 15% Multiple Intraventricular thrombi	Left CC CRI Bronchoscopy

AV—atrioventricular; ArV—arrhythmia ventricular (ventricular arrhythmia); AXIS—electrical axis; CAG—coronary angiography; CC—cardiac catheterization; CMD—cardiomyopathy; CMR—cardiac magnetic resonance; CRI—chronic renal insufficiency; CT—computed tomography; CXR—chest X-ray; DC—cardiac output; DI, aVL, V1–V6—electrocardiographic leads; ECG/EKG—electrocardiography; EF—ejection fraction; FE—fractional ejection (ejection fraction); GCM—giant cell myocarditis; HF—heart failure; HTP—pulmonary hypertension; ICD—implantable cardioverter-defibrillator; LBBB—left bundle branch block; LGE—late gadolinium enhancement; LV—left ventricle; LVEF—left ventricular ejection fraction; LVEFS—left ventricular ejection fraction (systolic); MRI—magnetic resonance imaging; MR—mitral regurgitation; NYHA—New York Heart Association; PET-CT—positron emission tomography–computed tomography; RBBB—right bundle branch block; RS—sinus rhythm; RT—tricuspid regurgitation; SSS—sick sinus syndrome; ST—sinus tachycardia; ST-T—ST-segment and T-wave changes; TS—sinus tachycardia; TSV—supraventricular tachycardia; TTE/ETT—transthoracic echocardiography; VT—ventricular tachycardia.

**Table 3:** Immunosuppressive treatment (IT).

Case No./Bibliography	Time Before the Diagnosis	Immunosuppressive Treatment (IT) Initiation	Immunosuppressive Treatment (IT) Maintenance	IT at Discharge
1 [16]	On admission	MP + IG iv	dual therapy	triple therapy
2 [17]	26 days	MP	monotherapy	
3 [12]	-	-	-	-
4 [18]	Immediately after admission	MP	dual therapy	
5 [19]	Untreated for GCM	-	-	-
6 [20]	44 days		dual therapy	same
7 [21]	-	-	-	-
8 [22]	22 days	IG + MP	Initial monotherapy then dual therapy	dual therapy
9 [23]	17 days	MP + ATG	triple therapy	
10 [24]	14 days	MP	dual therapy	
11 [25]	IT post-TC from day 38		triple therapy	same
12 [26]	>75 days	MP	dual therapy	
13 [27]	On admission	MP	dual therapy	same
14 [38]	16 days	MP + IG	dual therapy	same
15 [28]	34 days	MP	dual therapy	dual therapy
16 [29]	Roughly 60 days	dual drug therapy unfavorable development HT BAXILIXIMAB	Post HT triple therapy	
17 [30]	19 days	(1) MP (2) ATG + MP	Not mentioned	
18 [31]	On admission	MP + IG	monotherapy	
19 [32]	Relapse on admission after surgery		triple therapy	same
20 [33]	13 days	MP	monotherapy	monotherapy
21 [34]	11 days	rATG + MP	triple therapy	same
22 [34]	4 days	rATG + MP	triple therapy	same
23 [35]	2 days	MP	initial monotherapy iv with increase dose then triple therapy	
24 [36]	3 days	MP	Not mentioned	Not mentioned
25 [37]	<30 days	Not mentioned	triple therapy	Not mentioned

ATG—antithymocyte globulin; GCM—giant cell myocarditis; HT—heart transplantation; IG—intravenous immunoglobulin; IT—immunosuppressive therapy; IV—intravenous; MP—methylprednisolone; rATG—rabbit antithymocyte globulin.

### 3.4. Paraclinical Investigations Performed on the Study Group

#### 3.4.1. Laboratory Analyses

The following biological investigations were analyzed: troponin I, brain natriuretic peptide, and N-terminal brain natriuretic peptide. No correlation was observed between the intensity of the increase in the above-mentioned biological analyses, especially troponin, and the intensity of histological lesions, but, when corroborated by the clinical picture and other paraclinical investigations, especially ultrasound and MRI, PET-CT was an important tool in the initial diagnosis, in monitoring the evolution [16,28], and in guiding the therapeutic steps. As a result, plateauing at initially elevated values or an increase in these values under immunosuppressive therapy indicated the need to repeat the EMB and rethink therapy or was a warning sign for recurrence [16].

On the contrary, in some patients, despite the healing of histological lesions and improvement in general condition, troponin remained above normal values even after discharge [22].

In recurrent cases, this study group showed elevated troponin levels, but lower than during the initial episode of the disease [16].

- Troponin I/T (TnI): increases were observed in all 16 patients tested, with values ranging from 0.16 ng/mL to 14,210 ng/mL;
- N-terminal brain natriuretic peptide (NT-proBNP) was measured in nine patients, all of whom had elevated values ranging from 172 pg/mL to 53,482 pg/mL;
- Brain natriuretic peptide (BNP) was also elevated in all nine patients, with a minimum of 133 pg/mL and a maximum of 2170 pg/mL.

#### 3.4.2. Electrocardiography (ECG) (See Also Table 2, and Figure 1)

ECG was performed in 21 (84%) of the 25 patients, both at admission and during hospitalization. The ECG changes were nonspecific and heterogeneous, but, when corroborated by the clinical symptoms and other paraclinical investigations, they represented an important tool in the diagnosis of these patients.

**Figure 1:**
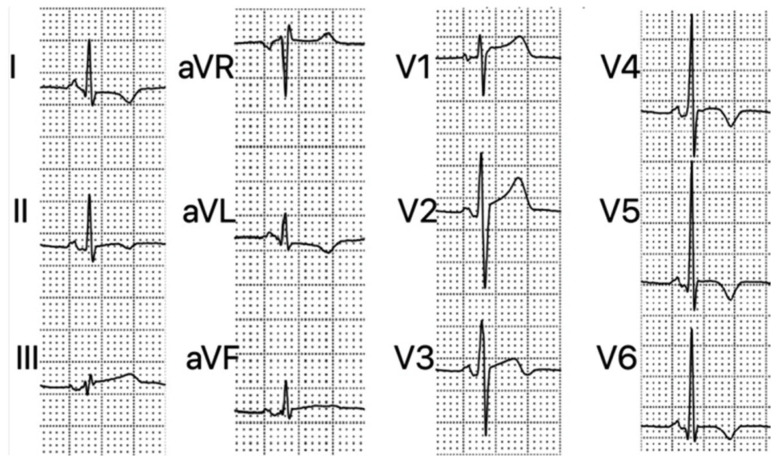
ECG findings in patients with myocarditis: PR segment depression in inferior and V2–V6 leads, PR segment elevation in aVR, ST elevation in inferior and V1–V3 leads, and T-wave inversion in I, II, aVL, and V3–V6 leads [39].

Thus, numerous ECG changes were diagnosed:

- Cardiac rhythm evaluation at onset: sinus tachycardia (ST) in 8 patients (34%), ventricular tachycardia in 3 (12%) patients [18,32], a ventricular bigeminy [19] with rapid progression to sustained VT or ventricular fibrillation (VF), refractory to treatment, followed by cardiogenic shock and cardiorespiratory arrest [12,13,14,15,33]; sometimes VT was sustained from onset [32].
- Cardiac conduction disorders were present at onset in 7 of the 20 patients investigated, ranging from first-degree atrioventricular block and partial bundle branch block with mild or asymptomatic symptoms to third-degree atrioventricular block [22] or complete left/right bundle branch block [12,23] and sick sinus syndrome requiring pacemaker implantation [24].
- Patients often presented with multiple rhythm and conduction disorders [12,23,25,26,35].
- Other ECG changes detected ST-T elevation in V2–V4 [17,25] with sometimes progression to marked changes in this segment accompanied by changes in the QRS complex [17], diffuse and nonspecific ST-T elevations [31], ST elevation in aVR and ST depression in aVL [35]; negative T waves in leads II, III, aVF, and right axis deviation.

#### 3.4.3. Transthoracic Echocardiography (ETT) (See Also Table 2 and Figure 2)

In this group of patients, this procedure was performed in 23 (92%) patients, revealing the following:

- Changes in cardiac kinetics in the left ventricle, ranging from mild hypokinesia with preserved LVEF [33] to severe global hypokinesia with LVEF below 10% and cardiac shock.
- In 17% of patients, biventricular involvement with severe hypokinesia and impaired ejection fraction of both the left and right ventricles was evident [18,23,29,36,37].
- A particular case was the detection of a space-replacing formation in the right atrium, accompanied by biatrial dilatation and preserved LVEF [24].
- Other lesions encountered were pericardial effusions [17,33,34,37], valvular insufficiency of varying degrees, septal hypokinesia [28], and concentric hypertrophy of the LV wall [34].

**Figure 2:**
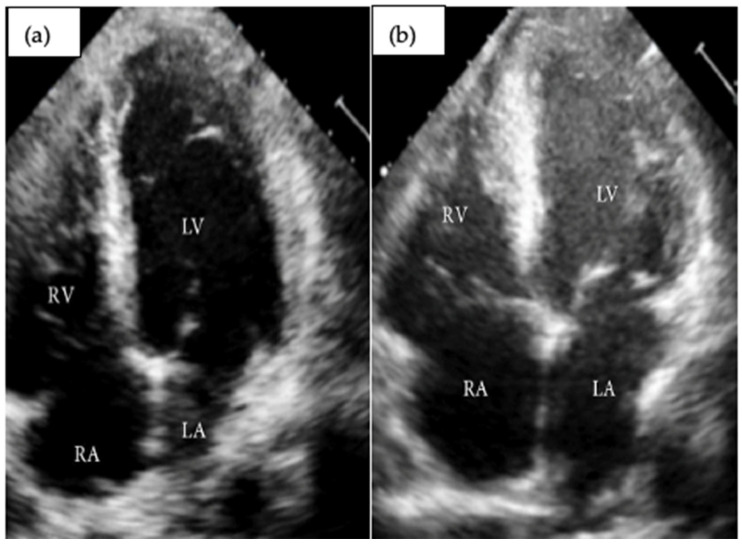
Apical four-chamber transthoracic echocardiography at admission (**a**) and after 7 days (**b**) showing reduced systolic function, increased wall thickness, and spontaneous echo-contrast present in the left ventricle (LV—left ventricle, RV—right ventricle, LA—left atria, RA—right atria) [40].

#### 3.4.4. Cardiac Magnetic Resonance Imaging (CMR) (See Also Table 2, and Figure 3)

CMR, a valuable imaging investigation, was performed in 10 (40%) patients upon admission, supplementing previous information for a definitive diagnosis or during the course of treatment and at periodic check-ups after discharge, along with other investigations for periodic monitoring.

**Figure 3:**
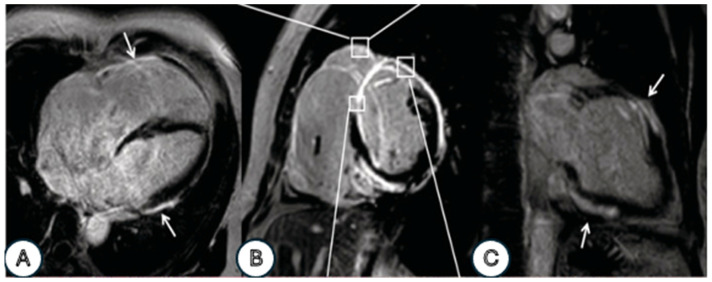
Cardiac magnetic resonance examination: late gadolinium enhancement (LGE) images of the short-axis (**B**), four-chamber (**A**), and two-chamber (**C**) views show an enhancing area in the right ventricle wall, transmural and both-sided LGE in the septum, transmural and epicardial LGE in the anterior wall, and epicardial LGE in the lateral and inferior walls (white arrows) [40].

Thus, upon admission, CMRI using gadolinium:

- Differentiated inflammatory cardiomyopathy from ischemic cardiomyopathy, detecting subepicardial hypersignal with a patchy transmural pattern in various locations, especially along the anteroposterior wall of the LV, consistent with myocarditis (subepicardial LGE) [18,19].
- Revealed subendocardial lesions specific to edema in the interventricular septum and anterior and lateral walls of the RV, with LGE present almost circularly in the LV and partially transmurally in the RV [14,28].
- Confirmed biventricular dysfunction detected on ETT [28].

T2-weighted MRI was used to highlight some inflammatory lesions, both at the cardiac level [15,32,34] and at the level of other systems and organs, such as the periocular muscles [16] or the back muscles [35].

In some cases, CMRI was performed during hospitalization, after renal function allowed it, in order to obtain the information necessary for a complete diagnosis and to see the evolution of cardiac lesions [18,22], and in other cases, after discharge, at periodic check-ups together with EMB [28,37].

#### 3.4.5. Coronary Angiography and Cardiac Catheterization (See Also Table 2)

Coronary angiography was performed in nine (36%) patients in this group, depending on the symptoms and changes detected in the other investigations, in order to rule out acute coronary syndrome.

In 7 of the 9 patients investigated, it was normal; in one patient, it detected nonspecific lesions, and in another patient who had type II diabetes and hypertension, it revealed moderate stenosis of the right coronary artery, with preserved blood flow [35] (see Table 2).

Cardiac catheterization, in addition to being used for biopsy sampling, in some cases complemented the changes detected on ultrasound, providing important and accurate information about the pressures in the heart chambers and large vessels, cardiac output, and the functionality and integrity of the heart structures. It was used both at admission for the most accurate initial diagnosis and assessment and during hospitalization to monitor cardiac parameters for rapid and effective adaptation of therapy.

#### 3.4.6. Anterior Cardiothoracic Radiography (CXR), Pulmonary Bronchoscopy (BP), Computed Tomography (CT), PET-CT and Fluorodeoxyglucose-PET (FDG-PET) (Table 2)

CXR was performed selectively in five (20%) patients based on their medical history, associated diseases, and clinical signs detected during physical examination; this revealed cardiomegaly and an image specific to pulmonary edema [12,35] in patients with dyspnea, productive cough, and crackles on physical examination.

BP was performed in one patient [37] for differential diagnosis with sarcoidosis.

CT was performed in 9 patients (36%):

- Initially upon admission, sometimes providing indirect information for the diagnosis of myocarditis [22] and thus guiding the final diagnosis or excluding some pulmonary pathologies such as pulmonary embolism or acute infectious pneumopathy [23];
- During hospitalization to exclude associated pathologies such as thymoma [35], completing information about vascularization and tumor formation in the left atrium [33]

FDG-PET or PET-CT was performed both during hospitalization to see the evolution of cardiac lesions under therapy [38] and after discharge at periodic check-ups [34].

#### 3.4.7. Endomyocardial Biopsy (EMB) (See Also Table 4 and Figure 4)

EMB remains the gold standard in the diagnosis of GCM. The histopathological findings of EMB in GCM typically show multifocal inflammation with giant cells, myocyte necrosis, and fibrosis [41]. In this study group, it was performed in 22 (88%) patients, sometimes upon admission but most frequently in the first month of illness.

**Figure 4:**
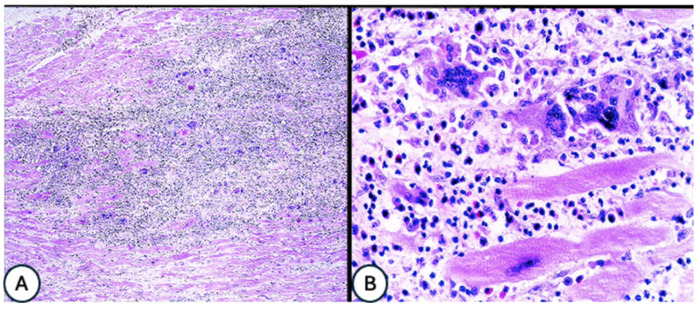
(**A**) Idiopathic giant cell myocarditis (IGCM) showing widespread necrosis of myocytes by a dense cellular infiltrate (H&E ×100); (**B**) high-power magnification of IGCM displaying giant cells, lymphocytes, histiocytic eosinophils, and damaged myocytes (H&E ×400) IGCM—idiopathic giant-cell myocarditis, H&E—hematoxylin and eosin stain [42].

There were also extremes in terms of the timing of the histological examination, namely:

- One puncture was performed more than a year after the onset of the disease and was negative for CMG, with the diagnosis being made 2 years after onset [19];
- In two cases, the diagnosis was made at autopsy [12,21];
- A patient with a heart transplant was diagnosed and treated without a puncture, as a relapse due to a history of GCM.

The first puncture was diagnostic for GCM in 18 (72%) patients.

EMB was repeated during hospitalization to assess lesions and response to treatment [35] in cases of suspected relapse [16,29] or to monitor the evolution of lesions under treatment after discharge [28].

**Table 4:** Endomyocardial biopsy (EMB) as the method of diagnosis.

Case No./Bibliography	Time Before the Diagnosis-EMB	First EMB Dgs GCM Yes/No	Number of EMB for Diagnosis
1 [16]	At admission	Yes	
2 [17]	26 days	Yes	
3 [12]	-	-	autopsy
4 [18]	Immediate	Yes	
5 [19]	2 years	No	2
6 [20]	45 days	Yes	
7 [21]	-	-	autopsy
8 [22]	22 days	Yes (the second reading)	
9 [23]	17 days	Yes	
10 [24]	14 days	Yes	
11 [25]	95 days	Yes	
12 [26]	>75 days	No	2
13 [27]	At admission	Yes	
14 [38]	16 days	Yes	
15 [28]	36 days	Yes	
16 [29]	13 days	Yes	
17 [30]	19 days	No	2
18 [31]	6 days	No	2
19 [32]	Without puncture		
20 [33]	Immediate after admission	Yes	
21 [34]	14 days	Yes	
22 [34]	7 days	Yes	
23 [35]	<7 days	Yes	
24 [36]	at admission	Yes	
25 [37]	<30 days	Yes	

EMB—endomyocardial biopsy, Dgs GCM—Diagnosis of giant cell myocarditis.

### 3.5. Immunosuppressive Treatment (IT) (Table 3)

IT was administered to 22 patients (88%), in most cases starting with 3 days of methylprednisolone pulse therapy (8 cases), with or without immunoglobulins (6 cases). (see Table 3).

Of the 22 patients, 8 underwent maintenance treatment with two immunosuppressant, using various combinations such as prednisolone and tacrolimus, prednisolone and cyclosporine, mycophenolate mofetil and tacrolimus. (see Table 3).

Triple immunosuppressive therapy was used in 9 patients:

- Patients who did not respond to the double combination [16,29,35];
- Patients with heart transplants, with or without recurrence in the transplanted heart [25,27,32] or in cases of corticosteroid resistance [35];
- Patients in serious general condition for whom the use of three immunosuppressants after pulse therapy was considered appropriate [23,34,37].

It should be noted that among the patients receiving triple combination therapy, two required the addition of a fourth immunosuppressant.

Prednisone monotherapy was administered to three patients.

Significant adverse effects included the following:

- Cholestatic hepatitis with azathioprine [22] was replaced with mycophenolate mofetil;
- Mycophenolate mofetil-induced pancytopenia [35] requiring discontinuation of administration;
- Severe thrombocytopenia with multiple etiopathogenesis requiring platelet transfusion [36].

### 3.6. Progression of Patients in the Study Group

The progression of patients was severe; of the 25 patients:

- Sixteen presented with cardiogenic shock upon admission or during the course of the disease, one of which resulted in death.
- Of the 15 patients with cardiogenic shock who survived, 11 required mechanical cardiovascular support during hospitalization and two were discharged with LVAD (see Table 5).
- Three patients had cardiorespiratory arrest (CRA), one of whom died following CRA.
- One undiagnosed patient died suddenly at home [21].
- Four patients underwent heart transplantation [19,25,29,30] and one is on the waiting list [23].
- Curative treatment or prophylaxis of recurrent arrhythmias with implantable cardioverter-defibrillator devices, LifeVest, or conduction disorders with pacemakers was performed in 7 patients, and one is scheduled for an implantable cardioverter defibrillator [20].
- Relapses were present in 3 patients, 2 of whom suffered relapses on the transplanted heart, with one patient suffering 3 post-transplant recurrences, with a very long period of hospitalization and changes in therapeutic regimens (Table 5).

## 4. Discussion

The literature states that giant cell myocarditis is a disease with an incompletely understood immune pathogenesis, rapid progression, and a frequently fatal outcome, predominantly affecting young and middle-aged adults [3,7].

**Figure 5:**
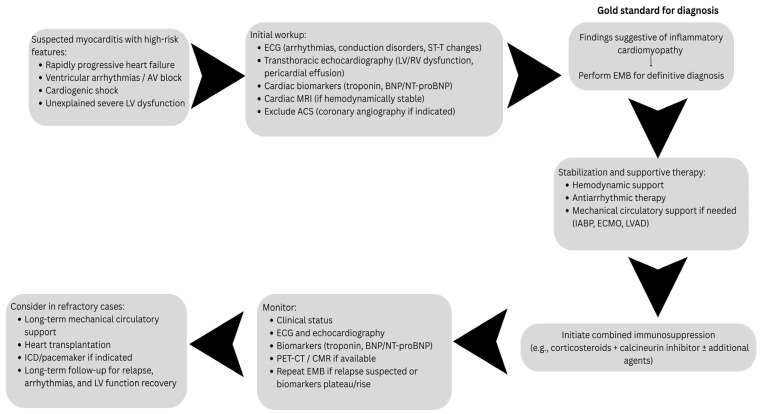
Proposed diagnostic and management algorithm for suspected giant cell myocarditis (derived from 25 reported cases).

Based on the diagnostic pathways, imaging strategies, biomarker profiles, and therapeutic approaches observed across the 25 analyzed cases, we propose a practical diagnostic and management algorithm (Figure 5) to assist clinicians in the early recognition and treatment of suspected giant cell myocarditis.

In our group of 25 patients, the average age was 44.92 years, with a minimum age of 22 and a maximum age of 76; there was no obvious gender predominance, with 12 men and 13 women, values close to those found in the literature [1,11].

The personal pathological history of the study group was divided as follows:

- Forty percent had autoimmune pathologies, with one patient being discovered at autopsy [21], while in the literature, the percentage was 19% [11].
- Twenty-eight percent had cardiovascular pathologies, of which 8% had a history of giant cell myocarditis, including one who had undergone heart transplantation.
- Eight percent of patients had undergone surgery for neoplasms.
- Twenty-eight percent of patients had multiple associated conditions (see Table 1).

As reported in the literature, the clinical picture at onset was polymorphic, with nonspecific cardiac symptoms. The most common form of onset was new heart failure, refractory to usual therapy, in which progressive dyspnea as a cardinal symptom was encountered in 48% of cases. The second most common form of onset, found in 20% of cases, was ventricular arrhythmias or cardiac conduction disorders, with palpitations as the clinical manifestation. Precordial pain of varying intensity dominated the clinical picture in 16% of cases, and general signs and symptoms and those of other organs outlined the onset of this condition in 12% of cases (see Table 2).

Although the onset modalities are nonspecific, the rapidity of symptom deterioration and the frequent progression to cardiogenic shock should raise suspicion of giant cell myocarditis, along with suspicion of other more frequent cardiac emergencies.

Troponin I/T, brain natriuretic peptide, and N-terminal pro-BNP tests were performed in some patients, but due to limited information about their evolution during the course of the disease and the low number of patients in whom they were performed, the final data were inconclusive. However, these laboratory investigations remain an important paraclinical tool in some cases of myocardial damage, their initial increase being an indication for further investigation even if the symptoms apparently improved [19].

There was no identifiable connection between the degree of histological myocardial injury and the levels of circulating troponin in the cases we examined. This finding is clinically relevant, as troponin is often used as an early marker of myocardial damage and disease severity in myocarditis [43]. Similar dissociations between biomarker levels and histopathological burden have been reported in other inflammatory cardiomyopathies, including lymphocytic and eosinophilic myocarditis, where troponin elevation may reflect the extent of acute myocyte necrosis rather than the overall inflammatory infiltrate or disease chronicity [44,45]. The variable and dispersed nature of inflammatory lesions in giant cell myocarditis may further impair systemic biomarkers’ capacity to appropriately represent the severity of the condition. These findings highlight the drawbacks of risk stratification based only on circulating biomarkers and the ongoing significance of multimodal imaging and endomyocardial biopsy in the diagnosis and prognostic assessment of suspected GCM.

The ECG changes were nonspecific, but their rapid onset and progression to a high degree, with severe symptoms, as well as their recurrence and resistance to standard antiarrhythmic therapy, corroborated by the other investigations, should raise suspicion of a diagnosis of GCM.

ETT remains the most commonly used, rapid, and accessible non-invasive investigation, providing initial information on cardiac kinetics, dimensional changes in the heart walls and chambers, and changes in heart valves, in terms of both structure and kinetics. Ultrasound changes have evolved rapidly, with a marked decrease in ejection fraction over a relatively short period of several weeks to a month, often leading to cardiogenic shock despite inotrope medication and the frequent need for mechanical cardiovascular support, which once again draws attention to a serious etiopathogenic substrate such as GCM.

CMI has provided valuable information about existing cardiac lesions and their stage of evolution, from active inflammatory lesions to pre-existing scars, differentiated between inflammatory and ischemic lesions, helping to rule out other serious conditions such as acute coronary syndrome, and provided detailed information about cardiac structure, function, and blood flow.

The wealth of information provided is sometimes offset by the contraindications existing in these patients. The serious general condition with marked dyspnea, cardiogenic shock, and the presence of a permanent pacemaker or other cardiac device make it impossible to perform this investigation. However, CMI remains an important investigation in these patients, being used both for the initial diagnosis to supplement previously detected information and sometimes to guide EMB, as well as for monitoring patients after discharge.

Other investigations such as cardiac catheterization, coronary angiography, CXR, BP, CT, PET-CT, and FDG-PET were performed in selected cases to provide valuable information for differential and stage diagnosis (CXR, CT, CC, Crf) and to monitor the evolution of cardiac lesions, highlighting myocardial metabolic activity (PET-CT/FDG-PET).

EMB remains the gold standard in the diagnosis of GCM. In this study group, it was performed in 22 (88%) patients, sometimes upon admission but most frequently in the first month of illness, with the first puncture being diagnostic for GCM in 18 (72%) patients.

The evolution of patients was generally severe, with 16 (64%) of 25 patients presenting cardiogenic shock, 4 (16%) patients dying, and 4 (16%) undergoing heart transplantation, for a total of 32% who died or underwent heart transplantation.

Conclusions regarding the evolution under TS are limited due to partial information (in some patients, even a lack of information) regarding the combinations used at discharge and the exact duration of maintenance treatment.

Despite the incomplete information, it can be said that TS is the basic treatment for healing histological lesions in CGM.

Current evidence indicates that giant cell myocarditis is an immune-mediated inflammatory cardiomyopathy characterized by T-lymphocyte–driven myocardial injury. This condition is frequently associated with autoimmune diseases and, in some instances, immune dysregulation resulting from infections or immune checkpoint inhibitors. The aggressive clinical course, including rapid progression to heart failure, malignant arrhythmias, and cardiogenic shock, likely reflects an uncontrolled immune response that leads to diffuse myocyte necrosis and inflammatory infiltration. These pathophysiological findings support the use of early and combined immunosuppressive therapy, which has been linked to improved transplant-free survival [46]. The standard treatment for GCM involves high-dose corticosteroids combined with a calcineurin inhibitor (cyclosporine or tacrolimus) and an additional agent such as azathioprine or mycophenolate mofetil. Induction therapy often consists of an intravenous methylprednisolone-a dose of 10 mg/kg (no more than 1000 mg/day) followed by oral prednisone with gradual tapering. After six to eight weeks, the prednisone dosage is often lowered from 40 to 60 mg per day to 5–10 mg per day. In addition, cyclosporine is given along with either alemtuzumab (15 mg/day for two days) or anti-thymocyte globulin (100 mg/day for three days). High-dose corticosteroids combined with cyclosporine and azathioprine (at a dose of 1.5–2 mg/kg/day) provide another possible therapeutic approach.

Careful monitoring of drug levels, blood counts, renal and hepatic function, and infectious complications is essential. Immunosuppressive therapy is typically continued for at least 12 months, although longer treatment may be required due to relapse risk [6].

The therapeutic regimen and duration of immunosuppressive therapy were individualized, being chosen according to certain parameters, such as:

- Pre-existing pathology of giant cell myocarditis;
- Severity of symptoms at onset and their evolution under the established therapy;
- Evolution of histological lesions under therapy;
- Age and adverse effects of therapy;
- The experience and intuition of the clinician.

Some patients underwent changes in their treatment regimen either due to adverse effects or an unfavorable initial response, which required the addition of a second, third, or even fourth immunosuppressant to the treatment regimen (see Table 3 and Table 5).

The timing of the initiation of immunosuppressive therapy, along with other factors such as age and personal medical history, influenced the outcome as follows:

- In the first week after the onset of the disease, immunosuppressive therapy was administered to 6 (24%) patients experiencing their first episode of GCM, with varying outcomes: a 70-year-old patient with diabetes mellitus and hypertension died of sepsis on the 146th day of hospitalization [36]; another 70-year-old patient with multiple comorbidities, who had undergone a liver transplant for autoimmune hepatitis and had chronic immunosuppression, survived and presented one year after discharge with an LVEF of 35% and NYHA II [18]; a 52-year-old female patient with associated diseases was discharged with an LVAD [36]; another 26-year-old with atopic diseases was well 6 months after discharge with no residual cardiac impairment [33], and the remaining two patients, aged 25 and 36, with no associated pathology, were well one year after discharge without immunosuppressive therapy [31,34] (Table 5).
- Patients treated more than 3 weeks after onset, young people under 35 years of age, without associated diseases, had a more severe progression; one underwent heart transplantation and three post-transplant relapses [29], and another was discharged with LVAD and planned for ICD-P in the future [20]. Another patient, aged 45 with no associated diseases, was hospitalized for over 80 days and underwent pacemaker and CRT-D implantation [22]. A 51-year-old patient with associated diseases died during a ventricular arrhythmia with cardiorespiratory arrest [17].
- Patients whose immunosuppressive treatment was administered between the second and third week after onset had different outcomes. At 22 years of age, without associated diseases and with monotherapy both as induction and maintenance, the outcome was good, with no relapses or sequelae [33], but at 69 years of age with associated diseases, a heart transplant was necessary [30] (see Table 5).
- There were also extreme cases, such as a 54-year-old patient with associated diseases who suffered multiple hospitalizations and a heart transplant and was diagnosed two years after onset.
- The fatal outcome observed—the 76-year-old patient [35] that died from candidal sepsis following multiple immunosuppressive regimens—highlights the “double-edged sword” of aggressive immunosuppression in giant cell myocarditis, particularly in elderly and critically ill patients.

While early and intensive immunosuppressive therapy is central to disease control and may improve short-term survival, it simultaneously increases vulnerability to severe opportunistic infections. This above-mentioned case underscores the need for careful patient selection, close infectious monitoring, and individualized risk–benefit assessment when escalating immunosuppressive therapy, especially in older patients with hemodynamic instability and prolonged exposure to invasive support. These considerations further emphasize the importance of a multidisciplinary approach involving cardiology, intensive care, infectious disease, and transplant teams.

Although supporting evidence is primarily extrapolated from studies of other non-ischemic cardiomyopathies, neurohormonal therapy is effective in managing heart failure, limiting cardiac remodeling, and improving long-term outcomes when administered in conjunction with immunosuppressive therapy. Neurohormonal therapy, which includes angiotensin-converting enzyme inhibitors, beta-blockers, and mineralocorticoid receptor antagonists, is recommended for patients with giant cell myocarditis (GCM) who have persistent left ventricular dysfunction [47]. Post-transplant evolution was variable: the 60-year-old patient with chronic supraventricular tachycardia was well with no relapses four years after transplantation [19], and a patient with common variable immunodeficiency and polymyositis had no relapse two years after transplantation [38]; both were treated with triple immunosuppressive combination therapy post-transplantation. A 33-year-old man with no previous medical history who underwent heart transplantation had three relapses post-transplantation under triple immunosuppressive therapy, as well as significant infectious episodes with prolonged hospitalization, finally adding a fourth immunosuppressant, with good subsequent evolution. In one patient, the post-transplant evolution is not mentioned.

Long-term follow-up data indicate that the risk of recurrence and major adverse events in giant cell myocarditis (GCM) persists well beyond the first year after diagnosis. In patients surviving more than one year without transplantation, histologically confirmed recurrence has been documented up to 8 years after initial presentation. Moreover, nearly half of long-term survivors experience death, transplantation, ventricular assist device implantation, or disease recurrence within five years. Recurrent heart failure, ventricular arrhythmias, renal dysfunction, and infectious complications are frequent during follow-up. Management of recurrence typically involves intensification or reintroduction of combination immunosuppressive therapy, which may stabilize disease progression [48].

### Limitations of the Study

Because giant cell myocarditis is uncommon and case-based reports predominate in the literature, the study is based on a very small and diverse cohort of 25 individuals. Because of this, the results are mostly descriptive and hypothesis-generating, and care should be taken when extending these findings to the larger group of individuals who may have myocarditis. Furthermore, the five-year period restricted the number of eligible cases, and incomplete follow-up in many publications made it impossible to objectively evaluate long-term outcomes, such as the ideal length of immunosuppressive treatment following discharge. Future prospective registries with standardized follow-up protocols are needed to better characterize the long-term course of giant cell myocarditis and treatment response.

## 5. Conclusions

In conclusion, the fate of patients with giant cell myocarditis has changed since the 1980s, when diagnosis was made at autopsy. Early diagnosis and rapid initiation of therapy remain decisive factors in mortality and morbidity in this condition. The prognosis remains serious, with an increased incidence of cardiogenic shock and malignant cardiac arrhythmias, but the early initiation of immunosuppression and the adaptation of treatment regimens as the disease progresses, as well as the modernization and superspecialization of some institutions in mechanical cardiovascular support and electronic devices such as cardioverter defibrillators and pacemakers, have significantly reduced the need for heart transplantation and, implicitly, post-transplant complications, as well as mortality from this disease.

Along with the rapid administration of immunosuppressive medication, the development of specific therapeutic regimens based on an accurate understanding of the pathogenesis of this disease and the risk factors for the disease and for an unfavorable evolution will lead to the effectiveness of this immunosuppressive therapy and a decrease in the incidence of immediate serious complications such as cardiogenic shock requiring mechanical support or malignant cardiac arrhythmias, as well as late complications such as recurrent GCM and arrhythmias or dilated cardiomyopathy, and an increase in the quality of life of patients.

## Figures and Tables

**Table 1:** General characteristics of the patients.

No Crt	Bibliography	Age/Sex	GCM in Personal Medical History/ Other Cardiovascular Diseases Associated	Autoimmune Diseases Associated	Neoplastic Diseases/Other Diseases Associated
1	[16]	58/M	GCM	ulcerative colitis polymyositis	
2	[17]	51/M		diabetes insipidus	
3	[12]	70/F		Hashimoto’s thyroiditis	breast carcinoma surgery
4	[18]	70/M	Atrioventricular block Bicameral pacemaker	liver transplant for AIH ulcerative colitis	colorectal carcinoma-colectomy
5	[19]	54/F	SVT		
6	[20]	33/M	-	-	-
7	[21]	36/M		psoriasis atherosclerosis pulmonary sarcoidosis	
8	[22]	45/F	-	-	-
9	[23]	62/F	-	-	-
10	[24]	39/M			acute pyelonephritis with bilateral hydronephrosis
11	[25]	32/F		polymyositis	CVID
12	[26]	56/F	-	-	-
13	[27]	26/M		ulcerative colitis atopic dermatitis myasthenia gravis	thymectomy
14	[24]	60/F	HF	hypothyroidism	
15	[28]	32/M	-	-	-
16	[29]	33/M	-	-	-
17	[30]	69/F	Non-ischemic dilated cardiomyopathy HF with DCM		
18	[31]	36/F	-	-	-
19	[32]	25/F	Cardiac transplant for GCM Post-transplant thrombosis of the left iliac artery and common femoral artery		
20	[33]	22/F	-	-	-
21	[34]	62/F	-	-	-
22	[35]	22/M	-	-	-
23	[36]	76/M	HTN		DM II
24	[37]	52/F		Sjogren syndrome	
25	[13]	27/M	-	ulcerative colitis	-

GCM—giant cell myocarditis; SVT—supraventricular tachycardia; AIH—autoimmune hepatitis; CVID—common variable immunodeficiency; HF—heart failure; DCM—nonischemic dilated cardiomyopathy; HTN—hypertension.

**Table 5:** Key points in disease progression according to age, presence of associated conditions, and immunosuppressive therapy, with references.

No. (Bibliography)	Gender and Age	Cardiogenic Shock	Cardiac Arrhythmias	Inotrope Medication	MCS	Important Highlights of the Patient Evaluation
1 [16]	M/ 58 years old	yes	-	Yes	-	- first relapse with cardiogenic shock under dual immunosuppressive therapy - persistent inflammatory lesions MMF added, good evolution with EF = 45% at discharge
2 [17]	M/ 51 years old	yes	VT 17 day of admission	Yes	Yes	- MP administered then monotherapy with prednisone unfavorable evolution -exitus
3 [12]	F/ 70 years old	SCA	AFib EV VFib + SCA	Yes	-	- in evolution FV and unresponsive cardiorespiratory arrest
4 [18]	M/ 70 an	yes	VA VT	Yes	-	- patient with DDD with good evolution under the triple therapy - one year after discharge, LVEF = 35%, NYHA II
5 [19]	F/ 54 years old	yes	Recurrent VT	Yes	Yes	- diagnosed 2 years after onset, when, being in cardiogenic shock, LVAD is implanted and - heart transplant at 60 years - 4 years after HT, with triple immunosuppression, no relapse or arrhythmias
6 [20]	M/ 33 years old	yes	-	Yes	Yes	- before starting IT, unfavorable evolution with cardiogenic shock with MSC - with IT has a relatively rapid but partial cardiac recovery, discharged with LVAD and double immunosuppression, also presented with headache and dizziness reason why ICD is planned
7 [21]	M 36 years old	-	-	-	-	- presented to the emergency department with nonspecific symptoms, was treated for SAD and SAV, died at home on the second day
8 [22]	F/ 45 years old		AV block pacemaker recurrent VT	yes	-	- under IT, double association and ICD-P for arrhythmia control - no relapse 6 months after onset
9 [23]	F/ 62 years old	Yes	-	Yes	-	- intubated, mechanically ventilated with shock refractory to standard therapy has a visible improvement in cardiac parameters after starting TS but remains with LVEF = 35% being a candidate for CT
10 [24]	M/ 39 years old	-	Refractory atrial flutter with bradycardia	-	-	- patient with surgically excised RA tumor formation - post-surgical atrial flutter - no initial response to double TS, which is why a DDD pacemaker is implanted with improvement of symptoms - 6 months after implantation, good progress
11 [25]	F/ 32 years old	Yes	-	Yes	Yes	- patient with CVID diagnosed with GCM, suffering from cardiac shock requiring IBPA - undergoes heart transplant with triple post-transplant TS - Grade II graft rejection at 11 weeks post-transplant - No signs of relapse at 2 years with the aforementioned triple combination
12 [26]	F/ 56 years old	Yes	-	Yes	Yes	- is treated with TS despite non-diagnostic EMB - progresses to cardiogenic shock with mechanical assist device despite treatment - no further details are provided about the progression
13 [27]	M /26 years old	Yes		Yes	Yes	- admitted with cardiogenic shock requiring MSC, treated with TS from admission and discharged with dual TS showing good progress 6 months after discharge
14 [38]	F/ 60 years old	Yes		Yes	Yes	- hospitalized with refractory IC suffering from cardiogenic shock, requiring MSC, - with relatively good evolution after TS is started - discharged with ICD, LVEF = 42%
15 [28]	M/ 32 years old	-	RBBB Nonsustained ventricular tachycardia	-	-	- initially receives monotherapy then dual immunosuppression is added due to asymptomatic TV appearing on EKG monitoring. - subsequent evolution is favorable.
16 [29]	M /33 years old	Yes		Yes	Yes	- unfavorable evolution with the need for LVAD and Right Ventricular Assist Device (RVAD) implantation despite dual TS association - underwent heart transplant, followed by 3 relapses of GCM with significant infections due to immunosuppression, being hospitalized for almost 3 years
17 [30]	F/ 69 years old	Yes	Recurrent ventricular arrhythmias	Yes	Yes	- The patient with Coxsackie B antibody titers has an unfavorable outcome with tow cardiogenic shock. - under TS, first require MCS. and second heart transplant,
18 [31]	F /36 years old	Yes	Recurrent VA	Yes	Yes	- The patient suffers from cardiogenic shock with - LVAD - has Echovirus 22 antibody titer - due to progression, GCM cannot be ruled out - administered 2 weeks of PS - one year after onset, doing well without TS
19 [32]	F/ 25 years old	Yes	Sustained ventricular tachycardia	-	-	- Patient with TC and triple immunosuppression and ICD-P presents shock at home detected by implanted defibrillator - considered GCM relapse due to history - increased dose TS, discharged in 2 days
20 [33]	F/ 22 years old	-	RBBB	-	-	- favorable response with PS monotherapy
21 [34]	F /62 years old	Yes	-	Yes	Yes	- with cardiogenic shock, shows improvement in parameters after TS - is discharged with triple TS, Life Vest, and ICD - 7 months after discharge, she is doing well
22 [34]	M /22 years old	Yes	VT VF with SCA	Yes	Yes	- initially suffered cardiorespiratory arrest but showed good cardiac parameter progression under TS initiation - 26 months after onset, no relapse or complications
23 [35]	M/ 76 years old	-	-	Yes	-	- elderly patient with associated diseases under TS presents persistent lesions on EMB and multiple adverse effects dies during candida sepsis
24 [36]	F/ 52 years old	SCA	VA with SCA	Yes	Yes	- Severe evolution with ACS,, receiving empirical pulse therapy in evolution, suffering ARF and HF - requiring LAVD and RVAD-ECMO, with good evolution - She is discharged with LVAD as long-term therapy - Maintenance TS not specified
25 [37]	M 27 years old	Yes	-	Yes		- with severe HF and cardiogenic shock, patients respond well to triple IT

CS—acute coronary syndrome; AFib—atrial fibrillation; ARF—acute renal failure; AV—atrioventricular; CVID—common variable immunodeficiency; DDD—dual-chamber pacemaker; ECMO-extracorporeal membrane oxygenation; EF—ejection fraction; EMB—endomyocardial biopsy; GCM—giant cell myocarditis; HF—heart failure; HT—heart transplantation; IBPA—IABP, intra-aortic balloon pump; ICD—implantable cardioverter-defibrillator; ICD-P—implantable cardioverter-defibrillator with pacing function; LV—left ventricle; LVAD—left ventricular assist device; LVEF—left ventricular ejection fraction; MCS—mechanical circulatory support; MMF—mycophenolate mofetil; MP—methylprednisolone; NYHA—New York Heart Association; PS—pulse steroid therapy; RA—right atrium; RBBB—right bundle branch block; RVAD—right ventricular assist device; SCA—sudden cardiac arrest; TS—immunosuppressive therapy; VA—ventricular arrhythmia; VF—ventricular fibrillation; VT—ventricular tachycardia.

## Data Availability

No new data were created or analyzed in this study.

## Abbreviations

AFib	atrial fibrillation
AIH	autoimmune hepatitis
AZA	Azathioprine
BAV	atrioventricular block
CC	cardiac catheterization
CAG	coronary angiography
CT	computed tomography
CYA	Cyclosporine
DM II	type II diabetes mellitus
EKG	Electrocardiogram
EMB	endomyocardial biopsy
ETT	transthoracic echocardiography
GCM	giant cell myocarditis
HF	heart failure
HT	heart transplant
HTA	Hypertension
HTN	Hypertension
ICD	implantable cardioverter defibrillator
ICM	cardiac magnetic resonance imaging
LBBB	left bundle branch block
MCS	mechanical circulatory support
MMF	mycophenolate mofetil
MP	Methylprednisolone
PS	Prednisone
RBBB	right bundle branch block
CXR	chest X-ray
SCA	sudden cardiac arrest
SVT	supraventricular tachycardia
TLS	Tacrolimus
TV	ventricular tachycardia
VA	ventricular arrhythmias
VF	ventricular fibrillation
VT	ventricular tachycardia
